# Assessment of perceptions, knowledge, and attitudes of parents regarding children’s schoolbags and related musculoskeletal health

**DOI:** 10.1186/s13018-019-1142-9

**Published:** 2019-04-27

**Authors:** Abdulmonem Alsiddiky, Raheef Alatassi, Fayez N. Alsaadouni, Khalid Bakerman, Waleed Awwad, Abdulrahman Alenazi, Sara Alsiddiqi, Husain Alyaseen

**Affiliations:** 1 Department of Orthopedics, College of Medicine, King Saud University, Riyadh, Saudi Arabia; 2 Department of Orthopedic Surgery, Security Forces Hospital, P.O. Box: 3643, Riyadh, 11481 Saudi Arabia

**Keywords:** Awareness, Knowledge, Back pain, Schoolbag, Child safety, Musculoskeletal

## Abstract

**Background:**

Heavy schoolbags and their adverse musculoskeletal effects are a cause of great concern. Parents’ awareness and knowledge about this are crucial to prevent such health problems. Thus, this study aimed to assess parents’ perceptions, knowledge, and attitudes regarding children’s schoolbags and related musculoskeletal health.

**Methods:**

A cross-sectional study was conducted targeting parents with children of school age. In January 2015, a self-administered, validated questionnaire was distributed to all participants, consisting of questions about their awareness, knowledge, and attitude regarding the negative effects of carrying inappropriate schoolbags on children’s musculoskeletal health.

**Result:**

A total of 616 parents (284 fathers and 332 mothers) completed the questionnaire (response rate of 100%). A total of 247 (87.3%) fathers and 301 (90.9%) mothers knew that carrying heavy schoolbags produces back problems. However, only 105 (36.9%) fathers and 107 (37.6%) mothers knew that incorrect schoolbag weight and inadequate way to carry it may impede the normal alignment and growth of the spine. Only 107 (37.6%) fathers and 96 (28.9%) mothers knew the ideal weight of the schoolbag, while 49.6% of fathers and 42.8% of mothers did not check their children’s schoolbags for unnecessary contents.

**Conclusion:**

Awareness of parents about the consequences of heavy schoolbags and correct use is still limited and suboptimal. Educational sessions for parents and awareness campaigns may help to reduce the prevalence of musculoskeletal health problems among children.

**Electronic supplementary material:**

The online version of this article (10.1186/s13018-019-1142-9) contains supplementary material, which is available to authorized users.

## Introduction

The incidence of use of schoolbags (backpacks) particularly among children of school age exceeds 90% in developed countries [1, 2]. Recently, several studies have shown that heavy schoolbags and their improper carriage for long periods of time may adversely affect children’s musculoskeletal system and not only produce fatigue and back pain, but also affect the normal growth of the spine [3–5]. Moreover, it may lead to abnormal spinal curves and even scoliosis [6–8]. Due to the high social and financial burden of back pain and spinal deformities, prevention is crucial. Despite the common belief, there is no true association with scoliosis till now.

Parents are the best judges and key persons to provide standard recommended schoolbags in terms of weight, size, straps, and waist support; additionally, they can also monitor how children carry the schoolbag and for how long. Furthermore, they can check for early symptoms such as back pain, abnormal posture, or spinal curvature in their children. Therefore, it is necessary to frequently assess parental awareness of the adverse effects of heavy schoolbags and knowledge regarding the recommended schoolbag weight, adequate type, and correct way to carry it. This will help to design interventional strategies to prevent schoolbag-related health issues among children [9–11].

In Saudi Arabia and many other countries, spinal deformities are not infrequent. The assessment of parental awareness of schoolbag-related musculoskeletal problems and implementation of campaigns and educational sessions during teacher-parent meetings will help to improve the health of children. Currently, there are few studies examining parental awareness of schoolbag-related problems worldwide, and even fewer in the region. In the present study, we aimed to assess the perceptions, knowledge, and attitudes of parents about children’s schoolbags and related health problems.

## Materials and methods

After an extensive review of the literature, a questionnaire (see Additional file 1.) was developed by the researchers to collect data on parents’ awareness of the use of schoolbags by their children. The questionnaire was intended to evaluate the following factors: awareness, knowledge, and attitude regarding children’s schoolbags and related musculoskeletal health issues. The questionnaire contained 19 questions divided into four parts.

The first part of the questionnaire collected participants’ demographic information, namely age, gender, education level, marital status, and salary. The second part contained questions on parents’ awareness of children’s schoolbags and related health issues. Parents’ knowledge about the recommended schoolbag in terms of load, straps, and carrying habits was assessed in the third part of the questionnaire. The questions in the fourth part examined parents’ practical attitudes toward reducing the negative effects of schoolbags on children’s musculoskeletal health. Additionally, their opinion on how to reduce health problems due to schoolbags in the future was also assessed. An initial evaluation of the questionnaire was conducted in a pilot study with a sample of 50 parents who were not involved in the main study. In addition, the questionnaire was reviewed by a group of psychologists, and orthopedic and spine surgeons. Simple revisions to the questionnaire were made in terms of clarity and simple wording based on the pilot-testing feedback.

The inclusion criterion was being a parent with one or more children attending school. The questionnaire was distributed among parents who attended an awareness campaign about schoolbags in different shopping malls in the city of Riyadh, Saudi Arabia, from 16 to 31 of January 2015.

### Statistical analysis

The IBM SPSS Statistics for Windows, Version 21.0 (IBM Corp., Armonk, NY), was utilized for statistical analyses. Frequencies and percentages were obtained for all variables. Chi-square or Fisher’s exact test were used to compare fathers and mothers with respect to different questions. The internal consistency of the questionnaire was tested using Cronbach’s *α*. A value of *p* < 0.05 was considered statistically significant.

## Results

A total 616 parents, including 284 (46.1%) fathers and 332 (53.89%) mothers, consented to participate and completed the questionnaire (100% response rate). Most participants were married (84.1%), while the remainder was either divorced (14.2%) or widowed (0.8%). A total of 63.1% were employed, while 9.1% and 18.9% were students and unemployed, respectively. Most of the parents lived in the city; however, a small number resided in villages. The monthly income of most of the participants was between 5000 and 20,000 SAR. Table 1 shows participants’ demographic data.

**Table 1:** Demographic characteristics of participants

Variables	Frequency *n* (%)
Parents	
Father	284 (46.1)
Mother	332 (53.89)
Marital status	
Married	525 (84.1)
Divorced	88 (14.2)
Widow	5 (0.8)
Employee	394 (63.1)
Freelance	50 (8.0)
Student	57 (9.1)
Unemployed	118 (18.9)
Illiterate	20 (3.24)
High school	158 (25.6)
University & above	438 (71.1)
Current place of residence	
City	587 (95.2)
Village	29 (4.7)
Income status	
Less than 2000 SAR	54 (8.76)
From 2000 to 5000 SAR	91 (14.7)
More than 5000 and 10,000 SAR	159 (25.8)
More than 10,000 and 20,000 SAR	219 (35.5)
More than 20,000 SAR	93 (15.09)

In general, 64% to 90% of parents had awareness about schoolbags and related health issues. However, the awareness rate for certain issues was very low. For instance, very few parents (36.9% fathers and 37.6% mothers) knew that heavy schoolbags carried for long time could produce spinal deformities in growing children. The awareness of mothers and fathers was about the same, with a small advantage of mothers. The data on awareness of parents regarding schoolbag-related health issues is presented in Table 2.

**Table 2:** Awareness of parents regarding adverse health effects of schoolbag

	Fathers	Mothers	*p* value
A heavy schoolbag can produce...			
Back pain	247 (87.3%)	301 (90.9%)	0.258
Neck pain	181 (64.2%)	247 (74.8%)	0.013
Shoulder pain	214 (75.9%)	286 (86.7%)	0.001
Spinal deformity	105 (36.9%)	124 (37.2%)	0.235

Table 3 shows the knowledge of parents regarding schoolbag issues. Only 107 (37.6%) fathers and 96 (28.9%) mothers knew about the recommended safe weight of the schoolbag. In addition, knowledge about how children have to carry the schoolbag was suboptimal.

**Table 3:** Knowledge of parents regarding adverse health effects of backpack

Do you know that...	Fathers	Mothers	*p* value
Weight of schoolbags should not be more than 10% of the child’s weight.	107 (37.6%)	96 (28.9%)	0.035
Schoolbags should have two shoulder straps rather than one.	247 (86.9%)	301 (90.6%)	0.258
Schoolbags should be carried on two shoulders and not on one.	181 (63.7%)	247 (74.3%)	0.013
Backpack-related official rules and penalties exist.	20 (7.04%)	24 (7.2%)	0.235

Regarding attitudes, the number of parents screening for schoolbag-related problems was very low. The majority of parents did not check the weight of their children’s schoolbag or ensure whether it is within the safe recommended range. Besides, most of the parents did not check for changes in their children’s posture, gait, or curvature of the spine to one side.

Table 4 shows parents’ opinions on how to reduce schoolbag-related health issues. Most of the parents agreed with reconsidering the curriculum and switching to e-learning.

**Table 4:** Opinions of parents on how to reduce health problems due to schoolbags

Opinions	Fathers	Mothers	*p* value
Redesigning the schoolbag	116 (40.8%)	117 (35.2%)	0.153
Redesigning the curriculum	180 (63.4%)	212 (63.9%)	0.903
Reducing amount of home work	64 (22.5%)	121 (36.4%)	0.0001
Employing a worker to carry student’s bags	16 (5.6%)	22 (6.6%)	0.610
Moving toward E-learning.	199 (70.1%)	206 (62%)	0.036

## Discussion

This study investigated parental awareness regarding schoolbags and related health issues. Herein, a suboptimal level of parental awareness regarding schoolbag characteristics and carrying habits was shown. Moreover, a wide gap exists between parental awareness and knowledge regarding the safe use of schoolbags and their actual practical attitude toward this issue. As a result, there is a need to bridge this gap with motivational and educational sessions for parents to avoid schoolbag-related health issues in children. Similar to the present study, some previous studies also found a suboptimal awareness and knowledge about this subject [10–12].

Since children have the closest relationships to their parents, parental awareness and supervision can ensure those of school age carry an adequate schoolbag. Additionally, they can teach them the correct way to carry the schoolbag on their shoulders, and monitor any adverse effects such back or shoulder pain. According to the results of the present study, more than two thirds of participating parents reported that they never checked their children’s schoolbags for unnecessary contents. Our results are in agreement with the findings of previous studies reporting similar parental attitudes [3, 10, 11, 13].

The recommended schoolbag weight that children should be allowed to carry it is about 10–15% of the child’s body weight [3, 14, 15]. The present study illustrated that the majority of parents did not know about this or the contribution of carrying a heavy schoolbag to spinal deformity.

According to the current results, most of the parents did not have sufficient knowledge about the signs and symptoms of musculoskeletal disorders resulting from incorrect use of schoolbags and the need to two shoulder straps. Additionally, awareness of the schoolbag carriage variables including recommended weight limit, appropriate method of carrying, and schoolbag strap adjustment was insufficient among the respondents in the present study. The findings also indicated non-significant gender differences in awareness of schoolbag use for the majority of questions. Fathers were more aware than mothers of the recommended weight limit and appropriate method of carrying schoolbags. Similar findings were reported in the previous literature [11–13, 16, 17]. Javadivala et al. [10] found that more than half of parents were not aware of the standard recommended weight or size of school backpacks.

Children’s musculoskeletal system grows mostly from 9 to 14 years of age. Abnormal stress on the spine due to carrying a heavy schoolbag not only produces back pain but may also cause a deformity in the developing spine. The wrong choice and use of backpack can lead to various health issues [6, 18–21].

Parents are responsible for their children’s health and thus in charge of promoting their safety. In this study, several recommendations for parents regarding appropriate schoolbag purchasing, content, and carrying methods are discussed and need to be followed. Namely, a schoolbag with double padded shoulder straps will help to absorb the load. Additionally, it is preferable to buy a schoolbag with a waist or chest strap, as this will help to keep the load of the bag close to the body and maintain a proper balance of the bag.

Regarding the schoolbag contents, parents must check daily what their children carry in the bag and ensure that they take only the items for their day’s activities and no unnecessary content. Regarding the carrying method, the two straps of the schoolbag must be worn over the shoulders. Hanging the schoolbag over one shoulder is not recommended because it may cause abnormal posture and leaning forward/sideways to compensate for the uneven weight, which can put abnormal stress on the spine. Over time, this can cause shoulder and back pain and even an abnormal curvature of the spine leading to scoliosis.

In our study, most of the parents agreed with reconsidering the curriculum and switching to e-learning in order to reduce schoolbag-related health issues. The results of this study will be helpful for government policy makers to launch regulations in favor of school children health and set a penalty for breaking such regulations. In addition, it is necessary to increase the awareness of parents, teachers, and children themselves about using appropriate schoolbags.

## Conclusion

Awareness of parents about heavy schoolbag consequences and correct use is still not sufficient. Our study indicates that while parents showed some awareness and knowledge of schoolbags and related health issues, there were considerable deficits in their knowledge about spine deformation due to abnormal schoolbag load and improper carrying. Furthermore, there were suboptimal attitudes toward checking children’s schoolbags for unnecessary contents, carrying textbooks according to schedule, and paying attention to children’s pains and posture. Most parents thought that reconsidering the curriculum and switching to e-learning would be helpful to reduce schoolbag-related health issues. Educational sessions and awareness campaigns for parents should be implemented to improve awareness and reduce musculoskeletal health problems among children.

## Additional file

Additional file 1: A questionnaire that used to collect data on parents’ awareness of the use of schoolbags by their children. The questionnaire contained 19 questions divided into four parts. (DOCX 14 kb)

## References

[1] Goodgold S, Corcoran M, Gamache D, Gillis J, Guerin J, Coyle JQ (2002). Backpack use in children. Pediatr Phys Ther.

[2] Whittfield JK, Legg SJ, Hedderley DI (2001). The weight and use of schoolbags in New Zealand secondary schools. Ergonomics.

[3] Brackley HM, Stevenson JM (2004). Are children’s backpack weight limits enough? A critical review of the relevant literature. Spine.

[4] Dianat I, Sorkhi N, Pourhossein A, Alipour A, Asghari-Jafarabadi M (2014). Neck, shoulder and low back pain in secondary schoolchildren in relation to schoolbag carriage: should the recommended weight limits be gender-specific?. Appl Ergon.

[5] Skaggs DL, Early SD, D’ambra P, Tolo VT, Kay RM (2006). Back pain and backpacks in school children. J Pediatr Orthop.

[6] Brackley HM, Stevenson JM, Selinger JC (2009). Effect of backpack load placement on posture and spinal curvature in prepubescent children. Work.

[7] Kim MH, Yi CH, Kwon OY, Cho SH, Yoo WG (2008). Changes in neck muscle electromyography and forward head posture of children when carrying schoolbags. Ergonomics.

[8] Korovessis P, Koureas G, Zacharatos S, Papazisis Z (2005). Backpacks, back pain, sagittal spinal curves and trunk alignment in adolescents: a logistic and multinomial logistic analysis. Spine.

[9] Whittfield J, Legg SJ, Hedderley DI (2005). Schoolbag weight and musculoskeletal symptoms in New Zealand secondary schools. Appl Ergon.

[10] Javadivala Z, Allahverdipour H, Dianat I, Bazargan M (2012). Awareness of parents about characteristics of a healthy school backpack. Health Promot Perspect.

[11] Forjuoh SN, Little D, Schuchmann JA, Lane BL (2003). Parental knowledge of school backpack weight and contents. Arch Dis Child.

[12] Dockrell S, Jacobs K, Byrne J, Gleeson E, Kelly S, Moore C (2017). Parental awareness of schoolbag carriage: a comparative study of Irish and United States parents. Work.

[13] Negrini S, Carabalona R (2002). Backpacks on! Schoolchildren’s perceptions of load, associations with back pain and factors determining the load. Spine.

[14] Malhotra MS, Gupta JS (1965). Carrying of schoolbags by children. Ergonomics.

[15] Wall EJ, Foad SL, Spears J (2003). Backpacks and back pain: where’s the epidemic?. J Pediatr Orthop.

[16] Cardon G, De Bourdeaudhuij I, De Clercq D (2002). Knowledge and perceptions about back education among elementary school students, teachers, and parents in Belgium. J Sch Health.

[17] Dianat I, Karimi MA (2014). Association of parental awareness of using schoolbags with musculoskeletal symptoms and carrying habits of schoolchildren. J Sch Nurs.

[18] Goh JH, Thambyah A, Bose K (1998). Effects of varying backpack loads on peak forces in the lumbosacral spine during walking. Clin Biomech.

[19] Rai A, Agarwal S, Bharti S, Ambedakar BBR (2013). Postural effect of back packs on school children: its consequences on their body posture. Int J Health Sci Res.

[20] Cavlak U, Cimbiz A, Akdag B (2006). Non specific low back pain in a Turkish population based sample of school children: a field survey with analysis of associated factors. Pain Clinic.

[21] Smith DR, Leggat PA (2007). Back pain in the young: a review of studies conducted among school children and university students. Curr Pediatr Rev.

