# Supplementary material for: Assessment of perceptions, knowledge, and attitudes of parents regarding children’s schoolbags and related musculoskeletal health
Source: J Orthop Surg Res. 2019 Apr 27;14:113. doi: 10.1186/s13018-019-1142-9 (PMC6487022; doi:10.1186/s13018-019-1142-9)
Supplement: Supplementary file 1 — A questionnaire that used to collect data on parents’ awareness of the use of schoolbags by their children. The questionnaire contained 19 questions divided into four parts. (DOCX 14 kb) [file 13018_2019_1142_MOESM1_ESM.docx]

**Additional file 1**

Participants’ demographic information:

1. Age
2. Gender
3. Education level
4. Marital status
5. Income salary

Are you aware that improper weight and improper carrying of schoolbags...

1. Can affect the muscles and spine and produce musculoskeletal health issues?
2. Can produce neck and shoulder pain and fatigue?
3. Can produce musculoskeletal low back pain?
4. Can affect the posture and cause spinal deformity?

10. Do you know that the weight of the schoolbag should not be more than 10% of the child’s weight?

11. Do you know that the schoolbag should have two shoulder straps rather than one?

12. Do you know that the schoolbag should be carried on two shoulders and not on one?

13. Do you know that prolonged bad posture can produce scoliosis (spinal deformity)?

14. Do you know there are backpack-related official rules and penalties?

Your attitude toward reducing the schoolbag weight:

15. Do you check the schoolbag of your child for extra contents?

16. Do you select the right schoolbag for your child, lightweight with waist and two shoulder straps?

17. Do you put textbooks according to the class schedule?

18. Do you regularly ask and check your child for muscular pains, fatigue, bending, and posture?

Your opinion regarding reducing health problems due to schoolbags:

19. What do you think is the best way to reduce health problems due to schoolbags?
